# Epigenetic regulations as drivers of insecticide resistance and resilience to climate change in arthropod pests

**DOI:** 10.3389/fgene.2022.1044980

**Published:** 2023-01-06

**Authors:** Kanakachari Mogilicherla, Amit Roy

**Affiliations:** Faculty of Forestry and Wood Sciences, Czech University of Life Sciences Prague, Prague, Czechia

**Keywords:** arthropod pests, insecticide resistance, climate change, epigenetic regulations, DNA methylation, histone modifications, symbiotic microbes

## Abstract

Arthropod pests are remarkably capable of rapidly adapting to novel forms of environmental stress, including insecticides and climate change. The dynamic interplay between epigenetics and genetics explains the largely unexplored reality underlying rapid climatic adaptation and the development of insecticide resistance in insects. Epigenetic regulation modulates gene expression by methylating DNA and acetylating histones that play an essential role in governing insecticide resistance and adaptation to climate change. This review summarises and discusses the significance of recent advances in epigenetic regulation that facilitate phenotypic plasticity in insects and their symbiotic microbes to cope with selection pressure implied by extensive insecticide applications and climate change. We also discuss how epigenetic changes are passed on to multiple generations through sexual recombination, which remains enigmatic. Finally, we explain how these epigenetic signatures can be utilized to manage insecticide resistance and pest resilience to climate change in Anthropocene.

## Highlights

❖ Epigenetics is a rapidly expanding research field addressing previously unknown gene regulation mechanisms.
❖ Epigenetics does not change the DNA sequence but reprograms gene expression in response to environmental stimuli like climate change and insecticide exposure.
❖ How epigenetic changes are passed on to multiple generations remains elusive.
❖ Epigenetic contribution to the rapid evolution of insecticide resistance and climate adaptation is poorly understood.
❖ We provide prospects to tackle pests impeding epigenetic mechanisms.
❖This review will interest molecular biologists, developmental biologists, entomologists, geneticists, and people involved in integrated pest management and readers of this journal.

## Introduction

Living organisms are influenced by environmental elements like temperature and light and ecological substances like phytochemicals and toxicants (Skinner, 2014a). Darwinian evolution relies heavily on the environment, and natural selection is a process in which environmental conditions affect the survival or reproductive success of individuals with various phenotypes (Darwin, 1859). The current paradigm in evolutionary biology holds that variations can evolve in response to natural selection due to changes in DNA sequence; however, phenotypic variation is thought to be due to genetic differences and is considered a neo-Darwinian process that the environment could alter through sociobiology-type mechanisms (Baldwin, 1896; Paenke et al., 2007; Laland et al., 2014). In neo-Darwinian evolution, the natural selection process is the primary mechanism through which the environment influences evolution (Olson-Manning et al., 2012; Laland et al., 2014). Combining heredity and evolution through natural selection seemed incorrect since it required an unacceptably high mutation rate to keep the trait variation needed for selection (Huxley, 1940). To answer this, Darwin suggested pangenesis, a sophisticated theory of transmitting environmentally sensitive somatic cells to progeny (Darwin, 1868).

It is not unexpected that climatic changes are altering the physiology, behaviour, abundance, and distribution of many species, given that global temperatures and precipitation patterns are changing quickly and profoundly impact living forms (Franks and Hoffmann, 2012; Good et al., 2016; Richard et al., 2019). The genetic impacts of climate change have not received as much attention as other climate change effects on the environment (Dawson et al., 2011; McMahon et al., 2011). As a result of recent findings in ecological genetics and developments in quantitative genetics and genomics, in our understanding, there are significant gaps in the likelihood and rate of adaptive genetic changes, the roles of regulatory and epigenetic effects, the evolution of plasticity in providing rapid selection responses, and more generally, the genetic architecture of adaptive evolution. Several shreds of evidence suggest that genetically based adaptive evolution traits, including body size, temperature responses, dispersion, and diapause/reproductive timing, may be a result of climate change (Thomas et al., 2001; Franks et al., 2007; Gardner et al., 2011; Hill et al., 2011; Karell et al., 2011; Richard et al., 2021). However, it is unclear whether numerous independently functioning genes govern the evolution of these traits with little influence or by a small number of essential regulatory genes found in genetic and metabolic networks. The exciting idea that heritable epigenetic modifications brought on by climate change could serve as an alternate and more rapid evolution method offers a captivating alternative to selection acting on existing genetic variation (Turner, 2009).

The ability of arthropod pests to develop resistance to insecticides and manage insect infestations is crucial nowadays, and insecticide expenditures in the world are approximately $40 billion each year (Pimentel et al., 1992; Popp, 2011; Bourguet and Guillemaud, 2016). Insecticide exposure in the field resulted in field-evolved resistance and reduced population vulnerability to insecticides (Tabashnik et al., 2014; Olivares-Castro et al., 2021). Individual events of insecticide resistance were better known through case studies, but the overall evolutionary process of insecticide resistance was still poorly understood (Gressel, 2011; Ffrench-Constant, 2013). Aggregating data from insecticide treatments on insect species to determine the trajectory of resistance could provide a road map for new techniques to combat resistance evolution. Observations of insecticide resistance evolution mechanisms in many insect species can provide useful insight into broader trends in resistance development within and between insect species. Most insecticide resistance research has focused on individual insecticide classes with biochemical or genetic mechanisms or the dissemination and growth of resistance in certain fields and species (Ffrench-Constant, 2013; Ffrench-Constant, 2014; Stratonovitch et al., 2014). These methods do not provide insight into the rate of insecticide resistance evolution, but they are critical for understanding how insecticide resistance evolves and spreads. Aggregated data and numerous methodologies were used for examining insecticide resistance development, highlighting larger evolutionary patterns across individual species and offering generalizable insights for pests with limited resources for thorough molecular or genetic studies (Brevik et al., 2018).

Resistance development is influenced by genetic, ecological, and operational factors, and the pace of insecticide resistance evolution varies significantly between species, yet the literature lacks systematic comparisons across geography and insecticide chemistries between species (Georghiou and Taylor, 1977; Council, 1986; Després et al., 2007). Although differences in the rate of evolution of species may explain evolvability, since some species evolve quickly, changes in evolvability differences against insecticides remain unexplained (Kinnison and Hendry, 2001). The evolvability of species is influenced by natural genetic variations such as differing mutation rates, biochemical differences caused by food variations, starting gene frequency differences, the number of generations each year, and population size (Carriére and Tabashnik, 2001; Lee and Gelembiuk, 2008; Hardy et al., 2018).

Insecticides and climate change alter insect development, physiology, and inheritance, and recent studies have focused on a link between insecticide and climate change effects on insects and their epigenetic regulations at the molecular level. Epigenetic mechanisms control gene expression without modifying genetic sequences against different stresses and compensatory mechanisms. This review summarises epigenetic changes from insecticide exposure and climate change on arthropod pests.

## Environmental epigenetics

**FIGURE 1:**
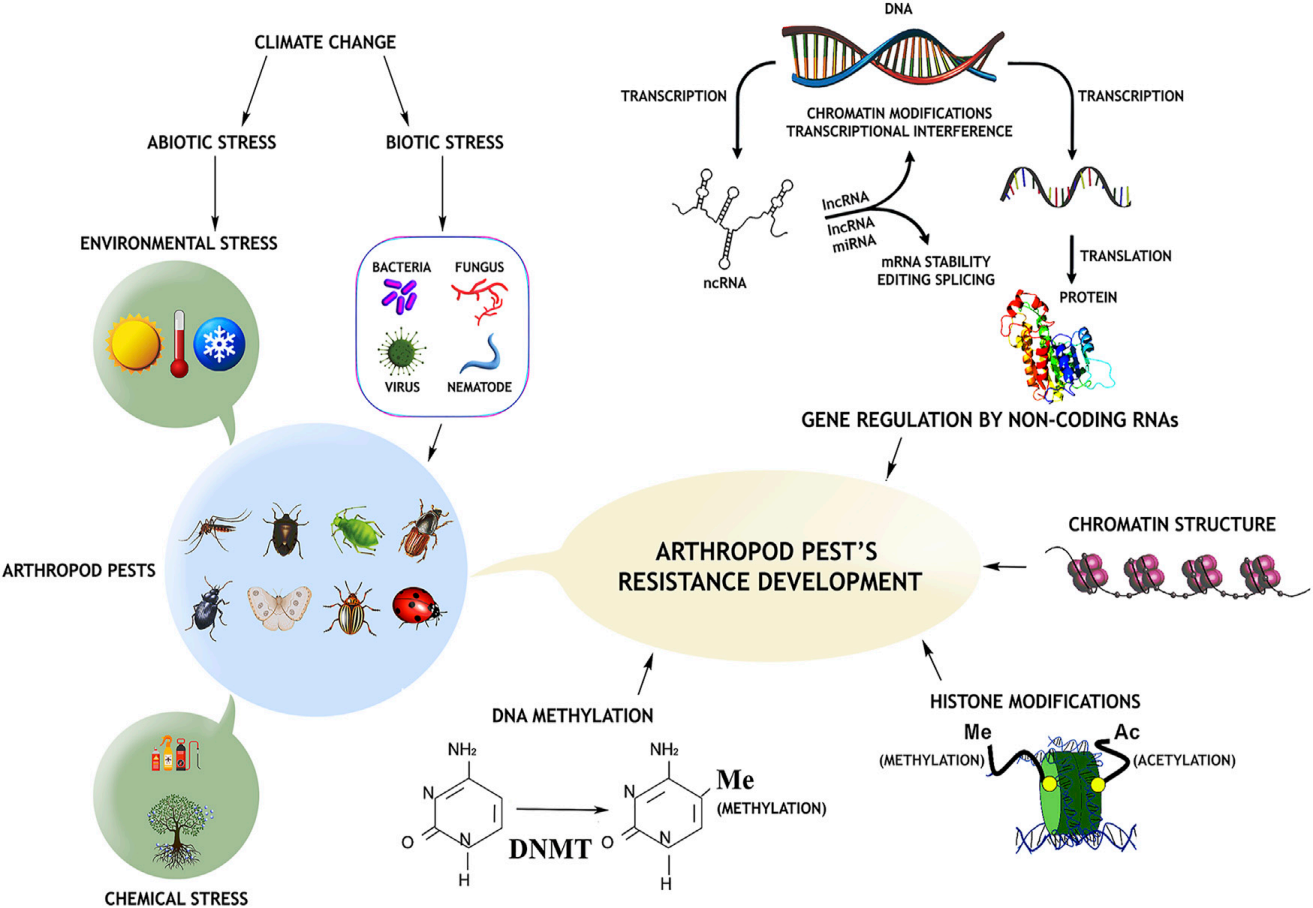
Arthropod pest resistance mechanisms emerge in response to environmental and insecticide challenges *via* epigenetic control. Epigenetic mechanisms include DNA methylation by DNA methyltransferase (DMNT) and histone modifications (acetylation and methylation). Furthermore, non-coding RNAs are also involved in gene expression alteration by modifying DNA or RNA. (snRNA- small nuclear RNA, lncRNA- long non-coding RNA).

The central idea of modern biology is Charles Darwin’s theory of evolution by natural selection, yet Darwin was unaware of the molecular mechanisms behind this process (Darwin, 1859). A robust neo-Darwinian theory of evolution was made possible by integrating Darwin’s ideas with developments in genetic and molecular sciences during the past century (Olson-Manning et al., 2012). Genetics and mutations are the current main theories for the molecular foundation of evolution, according to which genetic variations caused by chromosomal and random DNA changes directly impact phenotype and phenotypic diversity. Epigenetics is a biological mechanism that can significantly affect genome activity and contribute to phenotypic variance (Figure 1) (Skinner, 2011; Palli, 2021). The word “epigenetics” was first used by Waddington, and the traditional definitions are descriptive without considering the underlying molecular components (Waddington, 1953; Skinner, 2011). Epigenetic information controls how different cellular and organismal phenotypes are produced from the same genome, e.g., how insects can produce phenotypes suited to their habitats (Bonasio et al., 2010; Mukherjee et al., 2015; Glastad et al., 2019). Epigenetics focuses on heritable modifications to the regulation of genes in response to intracellular and extracellular environmental signals (Table 1) (McGuigan et al., 2021). Since many different stimuli can consistently alter gene regulation, epigenetic information can take many other forms as it is generally understood. However, molecular mechanisms that directly influence, modify, or interact with chromatin are often included under molecular epigenetics. Mitotic cell division within individuals and meiotic cell division, which results in progeny birth, can transmit epigenetic information. The development process and how an egg with a single set of genetic instructions can evolve into a multicellular organism made up of several tissues are addressed by intragenerational epigenetic inheritance (Waddington, 1942). The transmission of epigenetic information from a focused individual to offspring is the subject of intergenerational epigenetic inheritance (Heard and Martienssen, 2014). Intergenerational epigenetics, although assumed to be uncommon, is fascinating since it directly influences the course of evolution, and this distinction is crucial. According to current scientific knowledge, epigenetics is “molecular mechanisms around DNA that govern genome activity irrespective of DNA sequence and are mitotically stable” (Skinner, 2011). DNA methylation, histone modifications, chromatin structure, and specific non-coding RNA (ncRNA) are considered epigenetic mechanisms (Figure 1) (Skinner, 2014b). Pre and post-transcriptional gene regulations are the two types of epigenetic mechanisms. Before transcription, DNA methylation and histone acetylation/deacetylation occur, and post-transcriptional gene regulation occurs with small non-coding RNAs/microRNAs (miRNAs) (Figure 1) (Jaenisch and Bird, 2003; Gómez-Díaz et al., 2012). Recent reports also evaluated the influence of coding and non-coding RNAs in forest insect outbreaks (Zhang et al., 2020).

**TABLE 1:** Epigenetics studies in various insects during the last two decades.

S. No.	Insect order	Species name	Study	References
Environmental epigenetics				
1	Diptera	*D. melanogaster*	DNA methylation	Lyko et al. (2000)
2			In the fat body, miR-8 controls innate immune homeostasis	Choi and Hyun (2012)
3			Immune-responsive putative miRNAs have been discovered	Fullaondo and Lee (2012)
4			The link between histone acetylation and metabolism that extends the life	Peleg et al. (2016)
5		*D. pseudoobscura* and *A. gambiae*	Maintaining DNA methylation	Marhold et al. (2004)
6		*A. aegypti*	Wolbachia manipulates host gene expression and promotes colonization *via* using host microRNAs	Hussain et al. (2011)
7			Genome-wide patterns of cytosine methylation are disrupted by infection with a virulent strain of Wolbachia	Ye et al. (2013)
8			CREB-binding protein controls the development of compound eyes and metamorphosis	Gaddelapati et al. (2020), Gaddelapati et al. (2022)
9			To preserve the larval midgut, juvenile hormone-induced histone deacetylase 3 inhibits apoptosis	
10		*S. bullata*	Histone acetylation changes a pupal diapause	Reynolds et al. (2016)
11	Hymenoptera	*C. floridanus* and *H. saltator*	Caste-specific and genome-wide DNA methylomes	Bonasio et al. (2012)
12		*A. mellifera*	Alternative phenotypes, metabolic fluxes, gene splicing, and DNA methylation dynamics	Foret et al. (2012)
13			From egg to sperm, the dynamic DNA methylation cycle	Drewell et al. (2014)
14			An inhibitor of histone deacetylation controls the expression of genes involved in immunological signalling and detoxification	Hu et al. (2017)
15		*A. m. scutellate* and *A. m. ligustica*	The alternate splicing of mRNA and hydroxymethylation of intronic Non-CG DNA	Cingolani et al. (2013)
16		*A. m. ligustica*	The alternative splicing of the gene is affected by DNA methyl-transferase 3 knockdown	Li-Byarlay et al. (2013)
17		*N. vitripennis*	A comprehensive profile of DNA methylation	Beeler et al. (2014)
18	Hemiptera	*N. lugens*	For females to reproduce, DNA methyltransferases are crucial	
19		*S. furcifera*	Sexual dimorphism-related genomic regions with differential methylation	Zhang et al. (2015a), Zhang et al. (2015b)
20	Lepidoptera	*B. mori*	The methylome at a single base resolution displays a sparse epigenomic landscape	Xiang et al. (2010)
21		*G. mellonella*	During metamorphosis, injury, and infection, histone acetylation mediates epigenetic regulation of transcriptional reprogramming	Mukherjee et al. (2012)
22		*B. mori* and *B. mandarina*	Comparative methylomics between domesticated and wild animals suggests that domestication may have been influenced by epigenetics	Xiang et al. (2013)
23	Isoptera	*Z. nevadensis*	A termite’s genome contains clues of an alternate social structure	Terrapon et al. (2014)
24	Coleoptera	*T. castaneum*	The earliest proof of DNA methylation	Feliciello et al. (2013)
25			CREB-binding protein has been found to have several different roles throughout post-embryonic development	Roy et al. (2017), Roy and Palli (2018)
26			Acetylation and deacetylation of genes have been linked to the activity of juvenile hormones	
27			Involvement of CREB-binding protein in juvenile hormone activity	Xu et al. (2018)
28			Krüppel homolog 1 gene expression is suppressed by histone deacetylase 1, which also affects the activity of juvenile hormones	George et al. (2019), George and Palli (2020a), George and Palli (2020b)
29			Blocking the activity of histone deacetylase 11 prevents larval development and metamorphosis	
30			Histone deacetylase 3 is necessary for growth and transformation	
Insecticidal epigenetics				
31	Lepidoptera	*P. gossypiella*	Reversing the impact of transgenic insecticidal plants on insect adaption	Carriére and Tabashnik (2001)
32		*S. frugiperda*	Compared to laboratory-selected resistant strains, the gut bacteria of field-collected larvae are more varied and active in metabolizing a variety of pesticides	Gomes et al. (2020)
33		*P. xylostella*	PxABCG1 expression is regulated by the MAP4K4-controlled transcription factor POUM1, which affects Cry1Ac resistance	Xu et al. (2022)
34	Coleoptera	*L. decemlineata*	Cap ‘n' collar C controls the imidacloprid resistance-related genes	Gaddelapati et al. (2018)
35			DNA methylation patterns in subsequent generations are impacted by insecticide exposure	Brevik et al. (2021)
36		*T. castaneum*	Cap ‘n’ collar transcription factor controls several genes that code for proteins involved in the detoxification of insecticides	Kalsi and Palli (2017)
37	Diptera	*A. aegypti*	The gut microbiome is altered by permethrin resistance	Muturi et al. (2021)
38	Orthoptera	*L. m. manilensis*	Organophosphate resistance mechanisms in a field population	Yang et al. (2009)
39	Trombidiformes	T. urticae	Relationship between pesticide resistance and host plant adaptation	Dermauw et al. (2013)
40	Hemiptera, Lepidoptera, Coleoptera, Diptera, Thysanoptera and Hymenoptera	Different insects from different orders	The evolution of pesticide resistance is controlled by the pre-adaptation of a plant-eating insect’s diet	Hardy et al. (2018)
Transgenerational epigenetics				
41	Hemiptera	*M. persicae*	Loss of pesticide resistance is correlated with changes in DNA methylation	Field et al. (1989)
42		*E. heros*	Males’ sexual success following imidacloprid- and stress-induced hormesis	Haddi et al. (2016)
43	Hymenoptera	*A. m. carnica*	Genome-wide correlation between alternative splicing and DNA methylation	Flores et al. (2012)
44		*S. invicta* and *A. mellifera*	Histone alterations reflect the patterning and regulatory relationships of DNA methylation	Hunt et al. (2013b)
45	Coleoptera	*L. decemlineata*	Energy consumption, diapause behaviour, and possibility for northern range expansion	Piiroinen et al. (2011), Piiroinen et al. (2013), Piiroinen et al. (2014)
46			Pesticide stress is altered by the temperature stress experienced by earlier generations	
47			Exposure to deltamethrin has non-lethal effects on ancestors	
48			The synchronization of overwintering behaviour and physiology with photoperiod is necessary for northward range extension	Lehmann et al. (2014)
49	Diptera	*A. albopictus*	Epigenetic changes decrease insecticide sensitivity	Oppold et al. (2015)
50	Lepidoptera	*H. armigera*	HDAC3 knockdown shows dysregulation of juvenile hormone and apoptosis-related genes	Chang et al. (2022)
51	Different orders	Different insects from different orders	The role of intragenic DNA methylation and epigenomic insights	Hunt et al. (2013a)
Symbiotic microbiome epigenetics				
52	Diptera	*R. pomonella*	Degradation of insecticides by the bacterial symbiont *P. melophthora*	Boush and Matsumura (1967)
53		*A. aegypti*	Wolbachia controls methyltransferase and aids in the prevention of the dengue virus by using a host microRNA	Zhang et al. (2013)
54		*B. oleae*	Bacterial symbiosis overcomes host defences	Ben-Yosef et al. (2015)
55		*B. dorsalis*	The gut symbiont increases insecticide resistance	Cheng et al. (2017)
56	Hymenoptera	*A. m. mellifera*	Two insecticidal proteins and the reaction of the bacterial community in the intestines	Babendreier et al. (2007)
57	Hemiptera	*R. pedestris*	Resistance to insecticides caused by symbionts	Kikuchi et al. (2012)
58		*A. pisum*	Conserved small RNAs are expressed widely in tiny symbiotic genomes	Hansen and Degnan, (2014)
59	Aphelenchida	*B. xylophilus*	The symbiotic interaction in the degradation of xenobiotics is revealed by metagenomic research	Cheng et al. (2013)
60	Blattodea	Higher and lower termites	The diverse role of gut microbiota in digestion shaping the ecology and evolution of termites	Brune and Dietrich, (2015)
61	Coleoptera	*H. crudiae*, *H. eruditus* and *S. maurus*	The gut microbiome mediates caffeine detoxification	Ceja-Navarro et al. (2015)
62		*I. typographus*, *I. duplicatus*, *I. cembrae*; *I. sexdentatus*, *I. acuminatus* and *P. poligraphus*	Gut microbiome and their ecological significance in the bark beetle holobiont	Chakraborty et al. (2020a), Chakraborty et al. (2020b)
63	Lepidoptera	*P. Xylostella*	The *E. asburiae*, *B. cereus*, and *P. agglomerans* degrade acephate	Ramya et al. (2016a), Ramya et al. (2016b)
64			Possible involvement for carboxylesterase and esterase activity in the breakdown of indoxacarb in the culturable gut bacterial flora	

In DNA methylation, a methyl group to a cytosine residue in the dinucleotide sequence CpG results in 5-methylcytosine being added, which retains the base-pairing potential of unmodified nucleosides but changes its interaction with regulatory proteins (Figure 1). By methylating even a single CpG site in a promoter region, transcription of downstream genes can be reduced, and gene silencing can occur considerably (Robertson et al., 1995). In arthropods, cytosine methylation is vital in epigenetic gene control (He et al., 2015). DNA methylation has been discovered in several insect orders, including Coleoptera, Diptera, Lepidoptera, Hemiptera, Hymenoptera, Orthoptera, and Odonata (Table 1) (Xiang et al., 2010; Zhang et al., 2015a; Zhang et al., 2015b). DNA methyl-transferases (DMNTs) are enzymes that add a methyl group to specific chromosomal nucleotide bases of DNA and are classified into three families: DNMT1, DNMT2, and DNMT3 (Kausar et al., 2021). Because they are evolutionarily conserved, DNMT1 and DNMT2 are found in a wide range of insects, whereas DNMT3 is found exclusively in a few hymenopteran and hemipteran species, but the honeybee genome has all three DMNTs as well as a duplicated copy of DNMT3 (Glastad et al., 2011; Lyko and Maleszka, 2011). Using genome-wide methylation studies, scientists revealed the most prevalent DNA variation in insects such as *Nasonia vitripennis*, *Bombyx mori*, and *Apis mellifera* (Cingolani et al., 2013; Xiang et al., 2013; Beeler et al., 2014). Bisulfite sequencing can detect specific methylated genes, and CpG methylation was highest in *A. mellifera* and *Tribolium castaneum* embryos and decreased in the following stages of development (Feliciello et al., 2013; Drewell et al., 2014). According to genome-wide studies, insects have a significantly lower methylation to unmethylated CpG ratio than vertebrates, and less than 0.5% of the cytosine residues in the CpG dinucleotide are methylated in *Drosophila melanogaster* (Lyko et al., 2000; Marhold et al., 2004). In comparison to mammalian systems (60%–90%), invertebrates have very modest levels of CpG site methylation (0.36%–20%) (Regev et al., 1998; Suzuki and Bird, 2008). Many studies have found that DNA methylation is frequently restricted to genic regions of the genome (promoters, exons, and introns) rather than intergenic regions (Suzuki and Bird, 2008). Invertebrate insect genomes like hymenopterans (ants, bees, and parasitoid wasps) have lower amounts of DNA methylation than vertebrate genomes because methylation occurs at the promoters of genes and may inhibit transcription and metabolism (Ye et al., 2013; Yan et al., 2015). Several studies have suggested that gene splicing and activation are linked to DNA methylation in insects. Alternative splicing of mRNA transcripts caused by DNA methylation has been linked to behavioural control and caste specialization in eusocial insects (termites, bees, and ants) (Bonasio et al., 2012; Foret et al., 2012; Li-Byarlay et al., 2013; Terrapon et al., 2014). Furthermore, the removal of DNA methylation from the esterase gene of *Myzus persicae* reduced the transcription of this insecticide-detoxifying enzyme (FIELD, 2000). Recently, Gupta and Nair (2022) observed genetic plasticity in brown planthoppers and clarified the role of epigenomic modifications in converting environmental stimuli into heritable changes that may reduce stress and favor insects. They also showed how 5-azacytidine-mediated disruption DNA methylation influences the expression levels of some stress-responsive genes and highlighted demethylation/methylation as a phenomenon underlying rapid adaptive phenotypes (Gupta and Nair, 2022).

Histones are proteins found in the nuclei of eukaryotic cells that help to construct nucleosomes by packing DNA, and the positive charge of the core histones causes chromatin consistency and regulates transcription factor access (Figure 1) (Adcock and Lee, 2006; Bayarsaihan, 2011). Histones and wrapped DNA are densely packed in the condensed state of chromatin, making it transcriptionally inactive, but transcription factors could attach to DNA in the open chromatin and finally lead to gene expression. The addition or removal of acetyl groups from core histones increases or decreases DNA accessibility and thus promotes or suppresses gene expression, and histone acetylation and deacetylation are reversible epigenetic events that occur before transcription initiation (Marks et al., 2003; Hamon and Cossart, 2008). In insects, many biological and metabolic processes, such as growth regulation, development, metamorphosis, and reproduction, are regulated by juvenile hormones (JH) and ecdysteroids. The transcriptional co-regulators CREB binding protein (CBP) and Trichostatin A (TSA, HDAC inhibitor) activate several transcription factors that govern the expression of genes linked with post-embryonic development in insects. Histone acetyltransferases (HATs) and histone deacetylases (HDACs) are enzymes that acetylate and remove acetyl groups from amino acids of histone proteins, respectively. Three of the four classes of HDACs were found in *D. melanogaster* (Peleg et al., 2016). Our previous research demonstrated the importance of CBP and TSA for the regulation of HAT and HDACs during JH action in the red flour beetle, *T. castaneum*, and their cell lines and in yellow fever mosquito, *Aedes aegypti* (Roy et al., 2017; Roy and Palli, 2018; Xu et al., 2018; Gaddelapati et al., 2020). It has been demonstrated that there is a balanced upregulation of HATs and HDACs during insect metamorphosis in *Galleria mellonella* and *Sarcophaga bullata* (Mukherjee et al., 2012; Reynolds et al., 2016). In *G. mellonella*, an early pupal transition occurred in larvae treated with HAT inhibitors (suberonylanilide hydroxamic acid), and intriguingly, injection of HDAC inhibitors (sodium butyrate) delayed pupation. In addition, in the honeybee *A. mellifera*, sodium butyrate improved insecticide resistance (imidacloprid and microsporidian) by stimulating the production of immune and detoxification genes (Hu et al., 2017). Our previous lab investigated the function of class I and IV HDAC members (HDAC1 and HADC11) in *T. castaneum* and class I HADC3 in both *T. castaneum* and *A. aegypti*, respectively, and showed their effects on the acetylation levels of histones and the expression of genes coding for proteins involved in the regulation of growth, development, and metamorphosis (George et al., 2019; George and Palli, 2020a; George and Palli, 2020b; Gaddelapati et al., 2022).

MicroRNAs (miRNAs) are non-protein-coding short RNAs (18–25 bp) that can epigenetically upregulate or silence their target genes at the post-transcriptional level through either degradation of the target mRNA or translation inhibition (Bartel, 2009). They prevent the translation of mRNAs by base-pairing with untranslated sections or, in rare cases, the coding region (Ambros, 2004; Bartel, 2004; Asgari, 2011). MiRNAs are evolutionarily conserved across eukaryotic species and govern various cellular activities, including immunity, development, differentiation, and death (Bartel and Chen, 2004; Giraldez et al., 2005). Many studies have shown that a single miRNA can regulate hundreds of different target genes and influence more than 30% of animal genes (Bushati and Cohen, 2007; Bartel, 2009; Sato et al., 2011). Seven miRNAs were discovered in *D. melanogaster,* involved in the control of immunological responses (peptidoglycan receptor proteins), and another miRNA from the fat body was also discovered to influence immunity-related gene translation (Choi and Hyun, 2012; Fullaondo and Lee, 2012). Bacterial and viral infections have been linked to changes in miRNA expression levels in animals (Fassi Fehri et al., 2010). *Wolbachia pipientis* is an obligatory endosymbiont in a wide variety of invertebrates with the ability to manipulate host reproductive and host insect lifetime (Hussain et al., 2011). In addition, Wolbachia infection may also change the miRNA profile of the *A. aegypti* by inducing a host miRNA target metalloprotease gene, but, interestingly, inhibiting the miRNA reduces gene expression. To replicate efficiently in the host, the *Wolbachia* endosymbiont manipulates the host metalloprotease gene *via* the expression of cellular miRNA (Hussain et al., 2011).

DNA methylation is one example of an epigenetic process that can be imprinted and passed down to generations (Skinner, 2014b). It has been demonstrated that environmental factors encourage the epigenetic transgenerational inheritance of phenotypic variation. With epigenetics, the environment can directly modify phenotypic variation and its subsequent inheritance, e.g., DNA methylation must be removed to produce embryonic stem cells, causing the cell to become pluripotent (Crews et al., 2007; Seisenberger et al., 2013; Skinner, 2014b). Although many environmental influences cannot change the DNA sequence, temperature and nutrition can significantly impact epigenetic processes (Skinner, 2014a). All the examined organisms have highly conserved epigenetic mechanisms (such as DNA methylation) that are modifiable by the environment (Skinner, 2014a). A new molecular mechanism for controlling organism biology is provided by epigenetics. The environment’s capacity to directly affect cell and tissue development and function is essential for the individual’s health and phenotype, except in cases where the epigenetic alterations might be passed down across generations, and this direct environmental epigenetic effect on the individual would probably have little impact on evolution. Epigenetic transgenerational inheritance of disease and phenotypic diversity has been induced by various ecological variables, including temperature, nutrition, and environmental toxins (Skinner, 2014a). The germline transmission of epigenetic information between generations without direct exposure is known as epigenetic transgenerational inheritance (Skinner, 2011). Environmental exposures have imprinted epigenetic marks like DNA methylation during embryonic gonadal sex determination, gametogenesis, or another critical period of germline development (Skinner, 2014a). It has been demonstrated that factors like nutrition, body temperature, stress, and toxins encourage the epigenetic transgenerational inheritance of phenotypic diversity (Anway et al., 2005; Pembrey et al., 2006; Burdge et al., 2011; Song et al., 2013; Skinner, 2014a; Skinner, 2014b). Therefore, the ability of the environment to change the phenotype and alter phenotypic variation, independent of genetics, through this epigenetic mechanism is proposed to be essential for evolution (Anway et al., 2005; Jablonka and Raz, 2009; Day and Bonduriansky, 2011; Kuzawa and Thayer, 2011; Skinner, 2014a). Phenotypic variation is epigenetically transmitted through generations, and the environment can thus promote this process. Darwin cited sexual selection as one of the critical factors in evolution, and a prior study looked at how a hazardous environmental element could encourage the epigenetic transmission of a change in mate preference linked to sexual selection (Darwin, 1859; Crews et al., 2007). Therefore, an environmental element that changed sexual selection was discovered to encourage a long-lasting change in the sperm epigenome passed down through several generations (Crews et al., 2007). These findings suggest that ecological epigenetics may significantly influence evolutionary change (Skinner, 2011).

## Epigenetic regulations influencing insecticide resistance

Insecticide resistance has long been a concern affecting crop protection chemicals’ efficacy and usability, as well as firms’ capacity and inclination to invest in innovative crop protection compounds and traits globally (Sparks et al., 2021). In 1984, the Insecticide Resistance Action Committee (IRAC; https://irac-online.org/) was established to offer the crop protection sector a coordinated response to the problem of insecticide resistance and develop a few agrochemical businesses in Europe and the United States into a much bigger group of companies with global representation and an active presence encompassing corporations from more than 20 countries. IRAC also proactively addressed insecticide resistance management (IRM) by making various informational and educational tools (videos, posters, and pamphlets) on insect pests, bioassay methods, modes of insecticide action, and resistance management. The Insecticide Mode of Action (MoA) Classification Scheme, established by IRAC, has evolved from a relatively simple acaricide classification begun in 1998 to a significantly broader scheme that currently covers biologics as well as insecticides and acaricides. Since the discovery of insect resistance to insecticides, a growing number of insects have developed resistance to at least one or more of the insecticides available on the market (Feyereisen et al., 2015). Field-evolved resistance to insecticides is a genetically based reduction in a population’s susceptibility to an insecticide brought on by exposure to the insecticide in the field (Tabashnik et al., 2014). Inherent genetic variations, such as variable mutation rates between species, metabolic variations resulting from dietary variations, and starting gene frequency, may impact evolution (Lee and Gelembiuk, 2008; Hardy et al., 2018). Additionally, the number of generations produced annually and the size of the population may have an impact on the possibility that populations may develop resistance (Carriére and Tabashnik, 2001). The fact that pest species may be subjected to various amounts, chemistries, and rates of insecticides in entirely distinct environmental circumstances makes it challenging to compare the pace of insecticide development. For instance, a pest with a wide distribution may be exposed to a variety of insecticides regularly over several continents, whereas a pest with a more restricted distribution may only be exposed to a single chemical occasionally (Alyokhin et al., 2008; Yang et al., 2009). Arthropods have evolved defence systems in response to host plant allelochemicals (Figure 1). Similarly, they have created a variety of mechanisms, from behavioural to molecular to insecticide resistance (Heckel, 2014). Recent developments also identify transcription factors, internal microbial allies, and mechanisms underlying epigenetic and epitranscriptomics that promote insecticide and host plant resistance (Table 1) (Oppold and Müller, 2017; Gomes et al., 2020; Palli, 2020; Brevik et al., 2021; Muturi et al., 2021; Xu et al., 2022). Arthropods may regularly acquire insecticide resistance not just through novel mutations but also from existing genetic variations, suggesting that phytophagous arthropods have already pre-adapted to resist insecticides (Hawkins et al., 2019; Bras et al., 2022). Thus, if the chemical structure of the insecticides is like the plant defence chemicals, cross-resistance between host plant allelochemicals and insecticides could be a viable mechanism for rapid insecticide resistance development in arthropods (Després et al., 2007). Since the detoxifying mechanisms involved in host plant adaptation and insecticide resistance are similar, changes in host plant use may favour insecticide resistance and *vice versa* (i.e., pre-adaptation theory). This is supported by comparisons of genetic studies of host plant adaptation and insecticide resistance (Dermauw et al., 2013). Therefore, depending on their evolutionary history and the availability of existing genetic variation, some species may be more likely than others to evolve insecticide resistance (Bras et al., 2022).

In agroecosystems, insecticides are widely used and play a significant selective role in the evolution of insect pests, and the concept of the insecticide treadmill explains how insect pests develop a tolerance to routinely applied insecticides, rendering them useless (STAT, 2019). Based on mutation rates, the rapid gain and loss of resistance appear to happen far more quickly than expected, which raises the possibility that insecticides themselves can speed up mutation or alter the physiological makeup of pest species (Drake et al., 1998; Jablonka and Raz, 2009; Gressel, 2011). One potential justification that has not received much attention is that insecticide resistance may have evolved due to heritable epigenetic changes, which affect gene expression without altering the underlying DNA sequence. Insect pest species that have repeatedly encountered genetic bottlenecks owing to invasion and selection are nevertheless able to adapt relatively quickly, despite having low genetic diversity, which has led to the evolution of insecticide resistance (Gressel, 2011). The same insect pests have developed resistance to all the major classes of insecticides, and it is predicted that they will also develop resistance to new ones (Ffrench-Constant, 2007; Sparks and Nauen, 2015). Extreme genetic bottlenecks also don’t seem to be a barrier to the evolution of insecticide resistance. Insecticide resistance in pests appears inevitable because new phenotypes appear in response to environmental stress at rates that may not be explained by natural selection. Since environmental epigenetic patterning can affect the transgenerational transmission of phenotypic variation, it is crucial to understand how epigenetic processes fit within a neo-Lamarckian paradigm (Skinner, 2015). Xenobiotics and environmental stressors can directly affect the phenotypic responses of organisms to their environment through altering epigenetic changes. The study of epigenetics focuses on how the environment influences inherited alterations in gene expression. DNA methylation, histone changes, and heritable non-coding RNA are a few heritable epigenetic pathways that may be responsible for the transgenerational impacts of insecticides (Vandegehuchte et al., 2010b; Liebers et al., 2014; Niu et al., 2015). A heterodimer of xenobiotic transcription factors, cap n collar C isoform (CncC) and muscular aponeurosis fibromatosis (Maf), mediates cellular defence in invertebrates. These proteins in insects regulate the expression of genes involved in insecticide detoxification. We did RNAi and insecticide bioassays with insecticide detoxification genes in *T. castaneum* (CYP4G7, CYP4G14, GST-1, ABCA-UB, ABCAA1, ABCA-A1L, and ABCA-9B genes needed CncC for expression) and Colorado potato beetle, *L. decemlineata*, and discovered that CncC regulates genes coding proteins involved in insecticide detoxification (Kalsi and Palli, 2017; Gaddelapati et al., 2018).

## Epigenetic modifications, transgenerational inheritance, and response to xenobiotic stress

Epigenetic changes can be inherited (Vandegehuchte et al., 2010a). Most insect orders exhibit DNA methylation, which is the attachment of a methyl group to the 5-carbon position of the cytosine nucleotide (often the cytosine in CpG dinucleotides), an epigenetic inheritance mechanism that can affect phenotypic diversity (Figure 1; Table 1) (Glastad et al., 2011). In insects, methylation is primarily present in coding areas and is strongly related to gene expression and alternative splicing, which allows a single gene to produce a variety of gene transcripts with different lengths depending on which exons are translated (Flores et al., 2012). Methylation can occur anywhere in the genome, but the effects of DNA methylation differ depending on where it occurs: 1) changes in DNA methylation at the promoter region can affect gene expression in downstream genomic regions; 2) methylation suppresses the gene expression of transposable elements; and 3) gene body methylation can affect gene expression and increase the number of alternative splice variants (Field et al., 1989; Fablet and Vieira, 2011; Glastad et al., 2011; Hunt et al., 2013a). Methylation patterns in insects may change along with their levels of insecticide resistance (Hunt et al., 2013b). The nuclear DNA-wrapped histone proteins can be modified by adding acetyl or methyl groups, altering the regulation of gene expression (Szyf, 2015). Particularly among arthropods, the full ramifications of these alterations are not well understood, and it does seem that some histone changes can be passed down through generations (Holoch and Moazed, 2015). Various non-coding RNA (ncRNA) can be passed down by male or female gametes and sustain DNA methylation and histone modifications and thus influence the chromatin structure, while many recent studies have not looked at heritable RNA (Liebers et al., 2014; Peschansky and Wahlestedt, 2014). A phenotypic response results from a constellation of interrelated effects, including DNA methylation, histone changes, and ncRNAs (Vandegehuchte et al., 2009). It would be ideal to evaluate all three mechanisms simultaneously through concurrent small RNA-seq, bisulfate-treated DNA-seq, and histone modification assays in as many tissues or individuals as possible to fully understand how epigenetic modifications affect transgenerational phenotypic inheritance. To ascertain whether changes in epigenetics and gene expression are consistently different between treatments, it is ideal to sequence numerous generations, and these investigations are severely constrained by the cost of sequencing; however, anticipated decreases in sequencing prices in the future should make these studies possible.

Arthropods exposed to insecticides and other xenobiotic substances may experience alterations in their DNA methylation state, and these epigenetic modifications may last for at least a few generations (Vandegehuchte et al., 2010a; Vandegehuchte et al., 2010b; Poulos et al., 2017). A fungicide exposure causes heritable methylation alterations in mosquitoes and reduces their sensitivity to imidacloprid insecticides (Oppold et al., 2015). Higher rates of spontaneous deamination, which converts methylated cytosines to thymines in methylated areas than in non-methylated areas, can result in higher mutation rates (Baccarelli and Bollati, 2009). Genes that are most elevated in response to insecticide resistance may also be the most likely to experience spontaneous deamination if genes involved with resistance are methylated, which increases expression and increases the likelihood of mutation. Even though it has been demonstrated that DNA methylation and histone modifications frequently co-occur in the genome, it is less known how histone modifications and small RNA affect the epigenetic responses of arthropods to toxins (Szyf, 2015). Histone alterations allow for the epigenetically transmitted transmission of parental hormetic responses to oxidative stress to offspring (Kishimoto et al., 2017). Histone modifications can be altered by various environmental contaminants, including heavy metals, air pollutants, dioxins, and endocrine disruptors, although it is uncertain if these changes are inherited (Rechavi and Lev, 2017). Changes in small RNAs may also be related to the transgenerational inheritance of stress traits since small RNAs have been reported to interact with histone modifications (Piiroinen et al., 2011). A more recent study revealed that juvenile hormone (JH)-related and apoptosis-related genes were dysregulated by HDAC3 knockdown, and it came to the conclusion that the HDAC3 gene is a promising target for controlling *H. armigera* (Chang et al., 2022).

## Implications for transgenerational effects on insect fitness in agroecosystems

We propose that, through epigenetic mechanisms, insecticide use might, directly and indirectly, influence the evolution of insect pests in agroecosystems (Figure 1). The use of insecticides may directly promote the development of beneficial phenotypes that epigenetic changes may suppress (Table 1). Thus, continued insecticide application on populations encountering resistance would function as “natural selection” and systematically boost the prevalence of insecticide-adaptive insect phenotypes. Indirectly, the use of insecticides may maintain stressful surroundings so that hormetic prime insect pests can adapt to stressful situations more readily. For instance, sublethal insecticide exposure might alter adult body size, which may boost insect pests’ resilience to harsh winter circumstances (Alyokhin et al., 2009; Calabrese and Blain, 2011). Additionally, insecticides may improve female fertility or mate-seeking behaviour, resulting in a larger population (Piiroinen et al., 2014; Haddi et al., 2016). Phenotypic characteristics of pest insects that enable them to thrive when exposed to insecticides may also promote global invasions (Alyokhin et al., 2009). The insecticide-treated beetles invest more in fat bodies and have a greater metabolic rate than control ones (Piiroinen et al., 2013). Beetles may be better at detoxifying toxins because of their greater metabolic rates and larger fat bodies and can effectively overwinter using stored fat (Lehmann et al., 2014; Brander et al., 2017).

Contrarily, the intentional use of insecticides in agroecosystems is a component of an active pest management system, wherein insect responses to pressures can positively impact management choices in the future. As highly managed systems, agroecosystems enable more robust experimental control for studies at the field and landscape scales. Along these lines, it would be crucial to understand how individual, population, and species-level epigenetic responses to the same insecticides may vary. Such data would suggest whether epigenetic reactions can be broadly predicted across species and how insecticide resistance might be controlled more effectively. We may be able to understand better the role that epigenetic responses within insects play in the insecticide treadmill by combining new genomic techniques, epigenetic tests, and computationally demanding methodologies.

## Microbial influence on host regulatory mechanisms

Nowadays, insect-microbe interactions and the epigenome are among the most intriguing and perplexing fields of insect biological research, involving the symbiotic involvement of bacteria with their host organisms, particularly the gut-insecticide axis (Figure 1) (Gupta and Nair, 2020). Some of the most comprehensive and deep metabolic and ecological connections with gut flora are found in insects (Table 1) (Brune and Dietrich, 2015; Douglas, 2015; Kim et al., 2016; Mukherjee and Dobrindt, 2022). Our group recently expanded current understanding of core gut bacterial communities in *Ips* bark beetles and their putative functions, such as cellulose degradation, nitrogen fixation, plant xenobiotic compound detoxification, and pathogen inhibition, which could serve as a foundation for future metatranscriptomics, metaproteomics, as well as insect epigenomics research (Chakraborty et al., 2020a; Chakraborty et al., 2020b). In insects, the symbiotic connections mediated by DNA methylation appear to be rare, and only a few examples reveal strong methylation signals. One example is the symbiotic association between *Wolbachia*, an intracellular bacterium, and the mosquito *A. aegypti*. Infection with *Wolbachia* of the mosquito causes changes in host methylation because of impacts on the tRNA methyltransferase DNMT2, as well as immune system modifications that affect antiviral responses (Ye et al., 2013; Zhang et al., 2013). A massive number of non-coding regulatory RNAs, particularly sRNAs in archaea and bacteria, are likely to be more critical in insect-microbe interactions (Babski et al., 2014; Kim et al., 2016). Bacterial sRNAs can target eukaryotic genes, which can be coopted into the RNAi pathway or mimic eukaryotic sRNAs (Kim et al., 2016). Furthermore, bacterial symbionts with drastically reduced genomes have evolved several short RNAs that aid in modulating the expression of important symbiotic genes and regulating core housekeeping operations in their insect hosts (Hansen and Degnan, 2014). While evidence for microbe-mediated direct epigenetic impacts on organismal-level phenotypes in insects is limited, the potential for such regulation is considerable. Some insects (especially social insects) harbor enormous numbers of microbial species, each of which can potentially have some influence on complex regulatory functions (Engel and Moran, 2013). Future follow up studies in this area can reveal more exciting dimensions of inter kingdom interactions during insect symbiosis.

## Future perspectives and challenges

According to an emerging theory of environmental epigenetics, environmental exposure to a variety of chemicals can have long-lasting effects that are heritable, and the modifications have a variety of phenotypic impacts that last for generations, ranging from disease etiology to environmental change adaptations (Nilsson et al., 2018; Thiebaut et al., 2019; Sen et al., 2022). These epigenetic modifications can affect developmental bias, phenotypic flexibility, and niche creation, all of which influence the processes of evolution (Figure 1) (Jeremias et al., 2018). It is believed that epigenetic modifications brought on by the environment may have aided in the diversification and evolution of Darwin’s finches (Skinner et al., 2014). In arthropods, DNA methylation can be passed down across generations and affects important gene expression patterns, which may lead to enduring adaptation (Glastad et al., 2014). Additionally, alterations in DNA methylation are linked to modifications in vulnerability to insecticides (Field et al., 1989; Oppold et al., 2015). The rapid emergence of insecticide resistance can be explained in unique ways by understanding these mechanisms, which may also assist in addressing the enigma of insecticide resistance. One potential mechanism by which toxicity exposure reduces overall DNA methylation is due to competition between biological processes (Hunter et al., 2015; Oppold et al., 2015). The availability of methyl groups decides the DNA methylation of genomic DNA, and S-adenosylmethionine provides the methyl groups needed by methyltransferases (DMNT) to methylate DNA (Lee et al., 2009). Homocysteine, which is required as a precursor for S-adenosylmethionine, and glutathione are both antioxidants that conjugate with xenobiotic toxins (Enayati et al., 2005). Detoxification is necessary for the presence of toxins and depletes homocysteine, which may prevent the availability of S-adenosylmethionine for DNA methylation and result in a reduction in DNA methylation in the genome (Lee et al., 2009; Oppold and Müller, 2017). It is possible that the metabolic precursors required to methylate DNA are being depleted in this instance by the biochemical pathways involved in detoxification.

Designing novel chemical tools for insecticide resistance management in pest control requires a thorough understanding of the potential epigenetic mechanisms underlying insecticide resistance. Alternatively, the epigenetic system can most likely be used by insect molecular biologists and biochemists as targets for creating new insecticidal compounds (Jenkins and Muskavitch, 2015). Numerous epigenetic modifiers have been demonstrated to have a role in the control of developmental processes, homeostasis mediation, stimulation of responses to the external environment, and transmission of gene expression patterns through generations (Greer et al., 2011; Cantone and Fisher, 2013). Since epigenetic regulation genes are functionally conserved across all insect species, small molecule antagonists supplied in large quantities may also impact non-target insects (Jenkins and Muskavitch, 2015). Developing new and more effective insecticidal remedies targeting pest species depends on our ability to understand better epigenetic systems, mechanisms, and dynamics among insects. To understand the epigenetic dynamics in the insect gut microbiome related to climate change and insecticide resistance, scientists must concentrate on hologenomic research using cutting-edge laboratory techniques (metagenomes and CRISPR-Cas9 approaches). It will provide insight into how epigenetic modifications control the biomolecular characteristics of both host and microbiome communities (Kim et al., 2016; Gupta and Nair, 2020). Some studies demonstrated that insect resistance to biological (*Bacillus thuringiensis* and parasitoid wasps) and chemical insecticides (Fenitrothion) could be caused *via* symbiont-mediated processes (Oliver et al., 2003; Broderick et al., 2006; Kikuchi et al., 2012). However, the development of epigenetic analyses has challenges in establishing molecular techniques (ChIP-seq or ATAC-seq, etc.) to apply genome-wide chromatin dynamics analysis, revealing the epigenomic identity of alternative phenotypes. Instead of focusing on end phenotypes of insects, the studies will need to be focused on crucial stages (and potentially on specific tissues) when and where developmental switches occur and important symbiotic microbes. This will then allow for identifying critical variables that are receptive to environmental and insecticidal signals and will trigger cascades of gene expression alterations that eventually contribute to the formation of various resistance phenotypes. Finally, resistance management can only be effective if all stakeholders, including the crop protection sector, academia, regulators, crop consultants, growers, and other end-users, work together. IRAC was formed to raise resistance awareness among crop protection product developers, coordinate IRM tools and programs, forge alignment on key initiatives, and enable unified communication with other stakeholders. Thus, worries about insecticide resistance and reducing its impact remain paramount for crop protection companies, particularly those involved in discovering and developing new crop protection chemicals. IRAC and its linked IRM standards and programs are concerned with preserving the utility and efficacy of existing and novel crop protection, transgenic plant characteristics, and biological methods.

## Conclusion

Due to the widespread use of insecticides for insect pest management and unanticipated climate change, ecosystems can serve as excellent research subjects for studying epigenetic regulators of evolutionary and eco-evolutionary studies. However, the development of resistance also brought about conditions that were challenging to manage. More research on the epigenetic regulation underlying climate change and insecticide resistance evolution is needed to corroborate this pattern. Understanding the ecological and evolutionary consequences of insecticide resistance and climate change in arthropods and their symbiotic microbiomes will shed light on the potential effects of insecticides on multitrophic interactions and ecosystem functioning, which could aid in the development of more sustainable pest management methods (Figure 1; Table 1). To do this, it is critical to integrate a range of methods from molecular and quantitative genetics and genomics with ecological approaches for studying responses to climate change and insecticide resistance. The genetic underpinnings of insecticide resistance adaptation and climate change will be uncovered with recent developments such as genome sequencing, transcription profiling, metagenomics, metatranscriptomics, and bisulfite sequencing. In evolution, a better understanding of the genetic basis of adaptation to climate change and insecticide resistance in natural populations will address several issues, including the number of genes involved in evolutionary changes and their relative importance, the extent to which changes in gene regulation result in evolution, and the role of additional mechanisms, such as epigenetics. Based on existing research, our knowledge of the genetic underpinnings of evolutionary responses to climatic changes and insecticide resistance in arthropods and their symbiotic microorganisms is still limited (Table 1). We believe that the availability of insect genome sequences will remarkably induce epigenetic research on non-model insect pests. Research focused on non-model insects and their symbiotic microbes as holobionts against xenobiotics, insecticides, and climate change in Anthropocene using multi-omics approaches such as genome, transcriptome (coding and non-coding RNAs), metagenome, and metatranscriptome may aid in designing novel pest management strategies by impeding key regulatory mechanisms within pest insects in the postgenomic era.
